# Effects of a Single Session of High- and Moderate-Intensity Resistance Exercise on Endothelial Function of Middle-Aged Sedentary Men

**DOI:** 10.3389/fphys.2019.00777

**Published:** 2019-06-21

**Authors:** Francesco Pinto Boeno, Juliano Boufleur Farinha, Thiago Rozales Ramis, Rodrigo Cauduro Oliveira Macedo, Josianne Rodrigues-Krause, Jessica do Nascimento Queiroz, Pedro Lopez, Ronei Silveira Pinto, Alvaro Reischak-Oliveira

**Affiliations:** Program of Human Movement Sciences, Faculty of Physical Education, Physiotherapy and Dance, Universidade Federal do Rio Grande do Sul, Porto Alegre, Brazil

**Keywords:** resistance exercise, flow mediated dilation, endothelial function, nitric oxide, endothelin-1

## Abstract

Regular resistance exercise is associated with metabolic, neuromuscular and cardiovascular adaptations which improve quality of life and health. However, sedentary subjects have shown acute impairments in endothelial function after high-intensity resistance exercise. The aim of this study was to evaluate endothelial function in sedentary middle-aged men after a single session of resistance exercise at different intensities. Eleven sedentary middle-aged men (40.1 ± 3.9 years; 27.3 ± 1.4 kg/m^2^) underwent three different conditions of assessment: (1) single knee extension exercise at moderate intensity (MI) [4 sets of 12 repetitions at 50% of one repetition maximum (1RM) for each leg], (2) single knee extension exercise at high intensity (HI) (4 sets of 8 repetitions at 80% of 1RM for each leg), (3) resting for the control condition (CON). Flow-mediated dilation (FMD) was assessed before, 30 and 60 min after exercise. Plasma concentrations of endothelin-1 (ET-1), nitrites and nitrates (NO_x_) and thiobarbituric acid reactive substances (TBARS) were measured before, immediately after and 60 min after exercise. Blood pressure (BP) was measured prior to the experimental protocols, and in the following times: immediately following, and 2, 5, 10, 15, 30, and 60 min after exertion. There was a significant improvement in FMD 30 min after MI condition (12.5 ± 4.10 vs. 17.2 ± 3.9%; *p* = 0.016). NO_x_ levels were significantly higher immediately after MI (6.8 ± 3.3 vs. 12.6 ± 4.2 μM; *p* = 0.007) and there was a significant increase in the concentration of ET-1 immediately after HI (20.02 ± 2.2 vs. 25.4 ± 2.1 pg/mL; *p* = 0.004). However, there was no significant difference for BP (MI vs. HI) and TBARS among the experimental conditions. Resistance exercise performed at moderate intensity improved vasodilatation via increases on NOx levels and FMD in sedentary middle-aged men.

## Introduction

Physical exercise is related to decreased mortality due to cardiovascular diseases, contributing to the reduction of risk factors and the maintenance of endothelial function (Goto et al., 2003; Inaba et al., 2010; Green et al., 2011; McClean et al., 2015). In this sense, resistance exercise promotes well known significant adaptations to the neuromuscular system, such as increases in the functional reserve of its practitioners. Additionally, in recent years, resistance exercise has been emerging as a prevention and treatment strategy, eliciting positive effects on blood pressure (BP) regulation and insulin sensitivity (Williams et al., 2007).

Despite the aforementioned benefits, the effect of resistance exercise on endothelial function still needs to be clarified. Endothelium plays a crucial role in the regulation of vascular tone through the release of bioactive factors, such as nitric oxide (NO) and endothelin-1 (ET-1) (Shah, 2007; Green et al., 2011). The greater bioactivity of NO leads to antiatherogenic actions, which are triggered by an increase in endothelium-dependent vasodilation. On the other hand, high ET-1 levels are related to arterial hypertension and the progression of atherosclerotic disease (McEniery et al., 2002; Shah, 2007). It has been recently shown that during resistance exercise, the magnitude of ET-1-induced vasoconstriction increases with the aging process (Barrett-O’Keefe et al., 2015). In addition, high-intensity resistance exercise elevates ET-1 levels in sedentary individuals (Okamoto et al., 2008). This last effect seems to occur acutely, since resistance exercise chronically reduces ET-1 levels (Maeda et al., 2004).

In this sense, flow-mediated dilation (FMD) can provide a prognostic of endothelial function based in the concept that the direct assessment of the wall vessel response represents the endothelium behavior to environment through the release of vasoactive factors (Jurva et al., 2006; Green et al., 2011; Phillips et al., 2011; Franklin et al., 2014). Acute impairments of FMD, as well as elevations in ET-1 concentrations, may contribute to the lower bioavailability of NO and the increased incidence of cardiovascular events, especially in individuals with increased risk factors (Green et al., 2011; Thijssen et al., 2011; Dawson et al., 2013; Barrett-O’Keefe et al., 2015). Thus, the role of ET-1 may be a key to understand the endothelial function during resistance exercise. Additionally, the formation of reactive oxygen species (ROS) in response to higher intensities may attenuate endothelium vasodilatory response by decreasing NO bioavailability (Goto et al., 2003).

There is an acute impairment in the FMD of sedentary individuals after maximal resistance exercise, which seems to be related with a transient BP elevation after exercise (Phillips et al., 2011; Franklin et al., 2014). However, the cross-sectional studies aiming to investigate the FMD response to resistance exercise focused on large muscle groups and only used near-maximal protocols (above 90% of maximal load) (Jurva et al., 2006; Phillips et al., 2011; Franklin et al., 2014). It suggests that, in this population, higher intensities can lead to this phenomenon. However, studies involving different intensities of exercise, such as moderate resistance exercise, are needed to confirm this hypothesis. Indeed, exercise intensity can directly affect endothelial responses through some adjustments in blood flow pattern, metabolic demand and ROS formation (Goto et al., 2003; Thijssen et al., 2011; McClean et al., 2015).

Therefore, there is a need to understand the endothelial response to different intensities of resistance exercise, as well as the role of the endothelium-derived factor in the vascular tone after resistance exercise in sedentary people (Birk et al., 2012). Thus, the main goal of this study was to evaluate the acute effects of moderate- and high-intensity resistance exercise on FMD and plasma levels of ET-1, nitrites and nitrates (NOx) and thiobarbituric acid reactive substances (TBARS) in sedentary middle-aged individuals.

## Materials and Methods

### Participants

Eleven sedentary middle-aged individuals volunteered to participate in this study. The sample size was calculated, and subjects were recruited from the local community through the newspaper advertisement and social media. There were no dropouts during the study. Individuals who performed regular physical exercises, presented cardiovascular diseases or risk factors such as hypertriglyceridemia, hypertension, or diabetes were excluded. Additionally, to participate of the present study the individuals reported at least 6 months without any involvement in exercise programs. This study was approved of the local ethics committee (Permit No. 48788415.1.0000.5347).

### Experimental Design

This randomized cross-over study was composed by 5 days of evaluations separated by at least 72 h. Before every visit to the laboratory, subjects were instructed to perform 12 h of fasting, not to ingest alcoholic beverages or stimulants, not to perform physical exercises and to maintain the same food pattern 24 h preceding the tests. Evaluations were performed in the morning (7:00 to 8:30 a.m.) to minimize the effects of the circadian cycle on the variables evaluated.

On the first day of evaluations, subjects were submitted to a basal metabolic rate test, a blood test (fasting triglyceridemia and glycemia measurements), and 1RM test familiarization. On the second day of evaluations, the 1RM test was performed to determine the maximum dynamic force of the knee extensor muscles. The experimental protocols were randomly performed by drawing lots, by an independent person, considering the following conditions: high-intensity resistance exercise at 80% of 1RM (HI), moderate intensity at 50% of 1 RM (MI), or resting in the control condition (CON).

Upon arrival at the laboratory, individuals received a standardized meal equivalent to 15% of basal metabolic rate. 40 min after ingestion, subjects lay in the supine position to perform the following measurements at rest: blood pressure, FMD and blood tests (NOx, ET-1 and TBARS). Right after these resting measurements, the subjects randomly performed one of the three experimental conditions and returned immediately to the supine position. FMD was measured 30 and 60 min after the end of the exercise protocols. BP was measured before, right after and in the following times: 2, 5, 10, 15, 30 and 60 min after physical effort. Blood samples were collected before, immediately after, and 60 min after each experimental condition.

### Experimental Measurements

#### Basal Metabolic Rate

Basal metabolic rate tests were performed at 20°C–25°C, with controlled noise and low light. The protocol consisted of 10 min resting in the dorsal decubitus position, followed by 20 min of expired gasses collection. A computerized gas analyser (COSMED, Quark model CPET, Italy) was used to determine the VO_2_ and VCO_2_. To calculate basal metabolic rate (kcal/day), we used the equation proposed by Weir: [(3.9 X VO_2_) + (1.1 X VCO_2_)] x 1440 (Weir, 1990).

#### Maximum Dynamic Strength Test (1RM)

One repetition maximum (1RM) test was used to quantify the maximum strength values of the knee extensor muscles. Before the test, all subjects were previously familiarized with the equipment and with the procedures of the test (Konnen Gym, Beijing, China). The test was performed for knee extension exercise unilaterally, on both legs, with controlled 2-s metronome pacing for each phase of the exercise. Initially, 5 min of cycling at 15W was performed as warm-up for all the subjects. After warm-up, the subjects were positioned in the equipment and persuaded to start the test. If the individual could perform more than one repetition, a period of 3-min resting was respected between the attempts. Each individual had the maximum of five attempts to complete the test. 1RM was considered as the maximum load in which an individual could perform a repetition with good technique and full range of motion, followed by concentric failure.

#### Exercise Protocols

The experimental protocol was designed to mimic a typical resistance exercise set. In this way, the intensities and repetitions were selected based on the general guidelines (Williams et al., 2007). The experimental protocols were performed in a knee extension chair under three conditions: HI, MI, or CON. HI: the exercise was performed unilaterally, consisting of 4 sets of 8 repetitions at 80% of 1-RM for each leg. There was 2-min interval between the sets. MI: the exercise was performed under the same general conditions as HI, but the subjects were instructed to perform 4 sets of 12 repetitions at 50% of 1RM. In both conditions, all the subjects completed all reps without concentric failure. CON: the subjects remained seated in the equipment without making any effort for 15 min. Characteristics of the experimental protocols and participants are shown in Table 1.

**TABLE 1:** Sample characterization.

**Variables**	**Mean ± SD**
Age (years)	40.2±3.9
BMI (kg/m^2^)	27.3±1.5
Triglyceridemia (mg/dL)	114.9±25.8
Glycemia (mg/dL)	86.7±7
Basal metabolic rate (kcal/day)	1787±142
Rest systolic blood pressure (mmHg)	125.6±3.7
Rest diastolic blood pressure (mmHg)	82.4±3.2
**Maximum dynamic strength test (1-RM)**	
Right knee extensors (N)	627.6±143.2
50% of 1-RM (N)	313.8±74.5
80% of 1-RM (N)	502.1±120.6
Left knee extensors (N)	604.16±98.1
50% of 1-RM (N)	302±5.2
80% of 1-RM (N)	498.3±81.4

#### Arterial Flow-Mediated Dilatation (FMD)

The FMD analysis consisted of acquiring images of the brachial artery using a ultrasound system (Nemio XG, Japan) with a 7.5 MHz linear array probe. The position of the ultrasound probe was marked and measured according to the distance from the antecubital crease and all examinations were performed in the same position. FMD is an indirect measure of endothelial function and was adapted (Lopes Kruger et al., 2016), following previous guidelines (Thijssen et al., 2011). All evaluations were completed by the same blinded investigator with identical subject/equipment positioning.

The evaluations occurred in an air-conditioned room (21 to 24°C), always in the same period of the day, after 15 min of rest in the supine position. In the pre-occlusion period, five images were analyzed and the mean values were used as the baseline diameter of the brachial artery. After that, a pressure cuff placed on the forearm of the subjects was inflated to >240 mmHg, maintaining the pressure for 5 min. After 5 min of occlusion, the cuff was deflated and new images of the brachial artery were obtained with simultaneous electrocardiographic tracing. Three measurements were performed in each experimental condition (i.e., before, 30 and 60 min after the exercise protocol). All evaluation was recorded on DVD for further analysis in Image-J software.

In order to minimize the influence of the cardiac cycle on the arterial diameter, arterial thickness was determined always occurring in the “R” wave of the electrocardiogram (Thijssen et al., 2011). As a limitation of the technique, scans of the arterial diameter throughout the post-occlusion period were performed at fixed periods of 60, 90, and 120 seconds after cuff release. The mean dilation value found in each predetermined period was used for analysis. The values of FMD are shown as the percentage of increases in brachial artery diameter related to basal values. The percentage of vasodilation was calculated as follows:

$$\mathrm{Vasodilation}\%=\frac{{\left[\left(\mathrm{EH}-\mathrm{EB}\right)\right]}^{*}\,100}{\mathrm{EB}}$$

EH represents the dilation of brachial artery after reactive hyperaemia and EB the basal thickness of the brachial artery.

#### Blood Pressure

Blood pressure was determined by the auscultatory method using a properly calibrated mercury column sphygmomanometer with flexible cuff in the appropriate size, and a stethoscope (Littmann, Classic III, United States), as previously described (Franklin et al., 2014). Blood pressure was measured at rest, prior to the experimental protocols and at the end of the protocols at the following times: immediately following, and 2, 5, 10, 15, 30, and 60 min after exertion.

#### Dietary Control

Participants were instructed to avoid food rich in nitrites and nitrates 24 h prior to the sessions of assessment in order to minimize the influence of food intake in plasma levels of NO_x_. Additionally, a list of foods rich in nitrites and nitrates was provided for participants to know which foods to avoid. A standardized meal was provided prior to the experimental protocols, which was composed of 62% carbohydrates, 16% lipids, and 22% proteins. The energy content was calculated individually to correspond to 15% of the basal metabolic rate for 1 day. A sandwich composed of white bread with light cream cheese was offered.

In order to carry out the evaluation of the participants’ food consumption, a 24-h food recall (R24) was used. It was filled out as follows: the participant was asked about all food and drink consumed in the last 24 h before the performance of the three protocols (HI, MI, and CON). Data were calculated using the Diet Win Professional Nutrition Software (Brubins CAS, Brazil). Comparisons were made in between the content and quality of food consumed by the participants.

#### Blood Tests

Blood samples were taken from the antecubital region by a qualified professional, using sterile and disposable materials. Blood samples were collected into EDTA tubes and centrifuged at 3000 *g*. Samples were stored at −80°C for further analysis. Analysis of triglyceridemia and glycemia were performed through the colorimetric method using an automated analyser (Cobas C111, Roche, Switzerland). ET-1 levels were determined by enzyme-linked immunosorbent assay (ELISA) using commercial kits for humans (BosterBio, Pleasanton, CA, United States). NOx levels were determined through colorimetric method (Cayman, Ann Arbor, MI, United States). Analysis were performed in duplicate, according to manufacturers’ instructions. TBARS concentrations were analyzed as previously described (Ohkawa et al., 1979) and results expressed as μM of MDA/L. Results were obtained on a microplate reader (Thermo Fisher Scientific, Multiskan Go, Finland) at 532 nm.

### Statistical Analysis

Values are presented as mean ± standard deviation (SD). Normality of the data was tested by the Shapiro–Wilk test. A two-way analysis of variance (ANOVA) with repeated measures was performed to verify the effect of protocols and moments on the variables. A Bonferroni *post hoc* was used to locate the differences. Statistical significance was considered for *p* ≤ 0.05 and the Statistical Package for Social Sciences (SPSS 20, United States) software was used.

## Results

### Participants

Characteristics of participants and resistance exercise are shown in Table 1.

The standardized meal contained 268 ± 22 kcal. There were no differences in the macronutrient consumption among the three protocols. HI protocol: 1941 ± 695 kcal (carbohydrates: 54.1 + 10.6%; proteins: 22.2 + 7.6%; and lipids: 23.6 + 8.0%). MI protocol: 2170 ± 1055 kcal (carbohydrates: 53.6 + 9.0%; proteins: 21.0 + 7.5%; and lipids: 25.4 + 5.0%). CON protocol: 2076 + 861 kcal (carbohydrates: 52.5 + 11.4%; proteins: 19.8 + 4.6%; and lipids: 27.7 + 8.6%).

### Arterial Flow-Mediated Vasodilatation (FMD)

**FIGURE 1:**
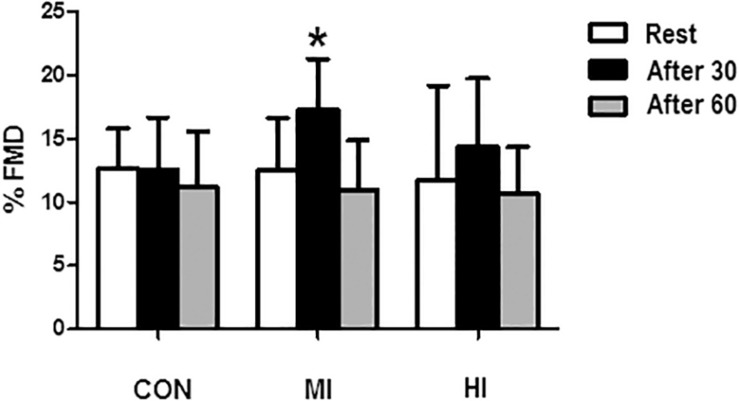
Percentage of brachial artery dilatation in response to different exercise conditions. ^*^Significant difference (*p* = 0.0016) in relation to pre-exercise moment for MI condition. CON, control condition; MI, moderate intensity; HI, high intensity.

There were no significant variation within-days in the diameter of the brachial artery before protocols (5.34 ± 0.6 vs. 5.46 ± 07 vs. 5.30 ± 0.5 mm, *p* > 0.05). However, a significant increase of FMD in the brachial artery was found 30 min after MI condition (12.5 ± 4.10 for 17.2 ± 3.9%, *p* = 0.01), returning to baseline levels 60 min after the protocol. No changes were found after HI or CON conditions. Changes in FMD are shown in Figure 1.

### Biochemical Variables

**FIGURE 2:**
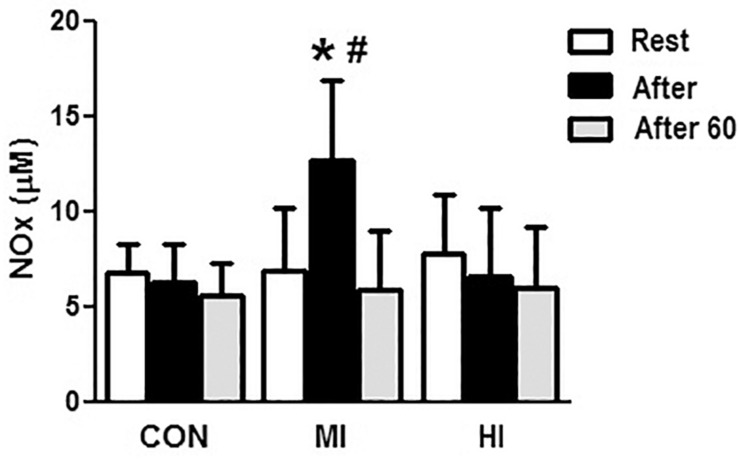
Concentrations of NOx in the plasma in response to different exercise conditions. ^*^Elevated plasma concentrations of NOx at post-exercise moment compared to pre-exercise moment for MI protocol (*p* = 0.007). ^#^Plasma concentrations of NOx significantly elevated at post-exercise moment for MI condition compared to HI (*p* = 0.015) and CON (*p* = 0.001). CON, control condition; MI, moderate intensity; HI, high intensity.

Concentrations of NO_x_ increased at the end of exercise for MI protocol (6.8 ± 3.3 vs. 12.6 ± 4.2 μM, *p* = 0.007). When comparing the different conditions, levels of NO_x_ were significantly elevated after exercise for MI condition compared to HI (12.6 ± 4.2 vs. 6.59 ± 3.62 μM, *p* = 0.01), and CON (12.6 ± 4.2 vs. 6.27 ± 2.22 μM, *p* = 0.001). No changes in NOx levels were found after HI or CON conditions. Figure 2 illustrates the concentrations of NO_x_ in the different protocols.

**FIGURE 3:**
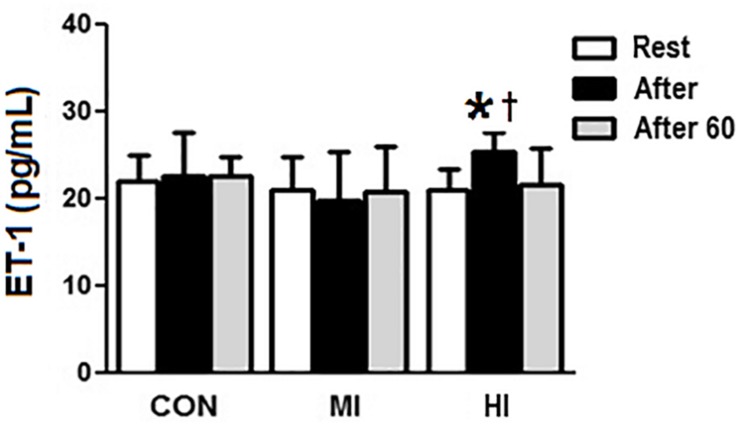
Plasma levels of ET-1 in response to different exercise conditions. ^*^ET-1 levels significantly increased (*p* = 0.004), compared to pre-exercise moment for HI condition. ^†^ ET-1 levels increased significantly at post-exercise moment for HI condition, compared to MI condition (*p* = 0.002). CON, control condition; MI, moderate intensity; HI, high intensity.

Concentrations of ET-1 increased significantly right after HI (20.02 ± 2.2 vs. 25.4 ± 2.1 pg/mL, *p* = 0.004), and decreased 60 min after exercise (25.4 ± 2.1 vs. 21.5 ± 4.1 pg/mL, *p* = 0.03). Moreover, there was a difference at post-exercise for HI when compared to MI condition (25.4 ± 2.1 vs. 19.8 ± 5.4 pg/mL, *p* = 0.002), but not when compared to CON (*p* > 0.05). MI and CON conditions did not induce to any significant changes on ET-1 concentrations (Figure 3).

No significant changes were found in plasma levels of TBARS (μM of MDA/L) for the different exercise conditions, as follows: CON: before: 1.34 ± 0.96, after: 1.12 ± 0.31, after 60: 1.13 ± 0.33. MI: before: 1.38 ± 0.27, after: 1.41 ± 0.24, after 60: 1.44 ± 0.29. HI: before: 1.26 ± 0.22, after: 1.18 ± 0.76, after 60: 1.59 ± 1.21.

### Blood Pressure

Both MI and HI conditions resulted in a significant increase in the absolute systolic blood pressure (SBP) in relation to the CON condition 2 min after exercise (HI: 174 ± 14 mmHg; MI: 158 ± 26 mmHg; CON: 124 ± 8 mmHg; *p* < 0.05). However, no significant differences were observed between MI and HI conditions. Diastolic blood pressure (DBP) did not change among time and conditions. There was no difference in SBP throughout the following measurements.

## Discussion

The main findings of this study are: (1) moderate resistance exercise significantly increases endothelium-dependent vasodilation 30 min after the session; (2) moderate resistance exercise increases NO_x_ levels immediately after the session; (3) high-intensity resistance exercise increases plasma levels of ET-1 immediately after exercise; (4) it seems there is no impairment to the vascular function (assessed by FMD) in response to MI or HI resistance exercise in sedentary middle-aged men.

Previous studies have shown that sedentary individuals present acute impairment in FMD after exercise (Gonzales et al., 2011; Birk et al., 2012; Dawson et al., 2013; McClean et al., 2015). Resistance exercise, specifically involving large muscle groups and performed at near-maximal intensity, is usually associated with the decrease of FMD, greater sympathetic activity and higher production of ROS (Jurva et al., 2006; Varady et al., 2010; Phillips et al., 2011; Franklin et al., 2014). Increased blood pressure levels afforded by resistance exercise appears to have a negative influence on FMD (Varady et al., 2010; Phillips et al., 2011). Therefore, we carried out the FMD assessments after the normalization of transient BP elevation (30 and 60 min after exercise) in order to minimize the influence of BP as a confounder.

It has recently been shown that exercise intensity influences FMD responses, particularly in protocols using hand grip (Atkinson et al., 2015) and cycle ergometer (Birk et al., 2012). This seems to be associated with the muscle size involved and the pattern of exercise-induced blood flow. Evidence has shown that retrograde blood flow leads to a marked decrease in FMD (Chappell et al., 1998; Himburg et al., 2007), while the anterograde flow increases the bioavailability of NO (Green et al., 2005; Tinken et al., 2009). In the present study, FMD was significantly increased only in MI. We speculate that MI induces a predominantly anterograde flow pattern, leading to increased bioavailability of NO. In fact, a significant increase in NOx levels for MI condition was found in our study. Moderate-intensity resistance exercise may induce to a laminar pattern of shear stress, stimulating the influx of endothelial calcium (Ca^2+^) (Olesen et al., 1988; Cooke et al., 1991), increasing the activity of endothelial nitric oxide synthase (eNOS) and consequently elevating NO bioavailability (Bussell, 2002). In agreement with our results, Willoughby et al. (2011) demonstrated elevation in NOx levels after exercise in similar conditions (Willoughby et al., 2011). As a matter of fact, elevated levels of NOx after exercise may represent a greater bioactivity of NO, because 85% of NOx plasma levels seem to be related to NO formation (Lundberg et al., 2009).

This study also demonstrates that high-intensity resistance exercise can elevate ET-1 levels. However, results regarding the modulation of ET-1 plasma levels in response to resistance exercise are very controversial. Otsuki et al. (2007) demonstrated that strength-trained individuals presented higher concentrations of ET-1 at rest when compared to their sedentary pairs (Otsuki et al., 2007). In the study by Okamoto et al. (2008), elevated ET-1 plasma levels were observed after hand grip exercise at 80% of concentric peak torque (Okamoto et al., 2008). On the other hand, Maeda et al. (2004) reported reduced levels of ET-1 after 8 weeks of strength training at 80% of 1 RM (Maeda et al., 2004). In fact, elevated ET-1 levels led to notable vasoconstriction and deleterious effects on blood vessels (Masaki, 2004; Shah, 2007; Thijssen et al., 2007). Moreover, McEniery et al. (2002) demonstrated that an infusion of BQ-123, an endothelin-1 type A (ETA) receptor antagonist, increased the vasodilator response to exercise (assessed by plethysmography) when compared to placebo (McEniery et al., 2002). These data suggest an important contribution of the ET-1/ETA vasoconstrictor mechanism not only in rest, but also in response to exercise.

We suggest two possible mechanisms that may have contributed to the increasing of ET-1 concentrations after the HI protocol in our study: (1) redirection of blood flow and (2) increased retrograde blood flow. During resistance exercise, the progressive activation of muscle fibers (quantify of fibers) is higher as the intensity increases (Okamoto et al., 2008). In this sense, higher muscle recruitment in the HI protocol increases demand of blood flow for active limbs. In this way, ET-1 secretion is elevated in the non-active limbs during exercise due to redirection of blood flow to active recruited muscles (Maeda et al., 1997). Changes in blood flow pattern that are agonistic for endothelin-converting enzyme (ECE) (Green et al., 2005) may have contributed to the elevation in ET-1 levels after HI. Indeed, there is evidence that increased retrograde blood flow following high-intensity exercise is associated with decreased FMD (Birk et al., 2012; Dawson et al., 2013; Atkinson et al., 2015). Thus, a possible retrograde pattern of blood flow may have induced to increases in ET-1 after the HI protocol. However, future studies are needed to confirm that.

It is well stablished that when connected to ETA receptor, ET-1 stimulates the opening of L-type Ca^2+^ channels, which remain open up to 60 min after stimulation (La and Reid, 1995). It is possible that this continuous entry pathway of intracellular Ca^2+^ limited the FMD response 30 min after the HI protocol. There is also an inverse relationship between NO bioavailability and ET-1 levels due to the inhibitory action of NO on ECE (Boulanger and Luscher, 1991). In the present study, no relationship was found between NO_x_ and ET-1 levels, however, high concentrations of NO_x_ and maintenance of ET-1 levels were found in MI. These results are consistent with the aforementioned evidence and with other studies that also demonstrated the inhibitory effect of NO over the ET-1 (Maeda et al., 2001; Otsuki et al., 2007).

Furthermore, impairments in endothelial function are systematically attributed to increases in ROS formation (McClean et al., 2015), mainly due to the formation of peroxynitrite. However, in our study, lipid peroxidation (evaluated by TBARS concentrations) did not change throughout the experimental conditions. These data are in agreement with Zembron-Lacny et al. (2008), which did not find changes in TBARS levels immediately and 24 h after resistance exercise (Zembron-Lacny et al., 2008). A possible mechanism for the maintenance of TBARS levels may be an acute increase in superoxide dismutase (SOD) and catalase (CAT) activity (Leaf et al., 1997). This may also indicate a maintenance of endothelial function in both experimental conditions, evaluated 30 and 60 min after RE. In addition, no changes were found in ET-1 and NO_x_ levels at 60 min after both conditions, concomitant with the normalization of FMD in this period.

There are some limitations to this study. We recognize that the assessment of oxidative stress by indirect markers only in plasma is a limitation of this study. Measurements of antioxidant enzymes in erythrocytes or leucocytes would be more accurate. Additionally, there was a significant variability regarding the resting FMD values within the HI group, which could have contributed to the lack of significant difference observed in this condition. Finally, the blood flow analysis, as velocity and pattern, could contribute to add information to the present study.

## Conclusion

To the best of our knowledge, this is the first study to analyze endothelial function in response to different intensities of resistance exercise (using large muscle groups) through FMD and vasoactive factors derived from the endothelium. Our results demonstrate that moderate-intensity resistance exercise can increase FMD, along with elevations in plasma NOx concentrations, in middle-aged sedentary men. We also showed that high-intensity resistance exercise (80% of 1RM) lead to increases in ET-1 concentrations. However, future studies are needed to investigate the actual contribution of the vasoconstrictor mechanism of ET-1 to FMD. Finally, longitudinal studies purposing resistance exercise training at moderate intensity would help to clarify chronic endothelial adaptations to this type of exercise in sedentary individuals.

## Ethics Statement

All the experimental procedures of this study were carried out in accordance with the internationally accepted standards for human research and approved by the Ethics Committee of the Universidade Federal do Rio Grande do Sul.

## Author Contributions

FB, JF, TR, RCM, JR-K, JNQ, PL, RSP, and AR-O contributed to the design and implementation of the research, analysis of the results, and writing of the manuscript.

## Conflict of Interest Statement

The authors declare that the research was conducted in the absence of any commercial or financial relationships that could be construed as a potential conflict of interest.
